# Free-living gait characteristics in ageing and Parkinson’s disease: impact of environment and ambulatory bout length

**DOI:** 10.1186/s12984-016-0154-5

**Published:** 2016-05-12

**Authors:** Silvia Del Din, Alan Godfrey, Brook Galna, Sue Lord, Lynn Rochester

**Affiliations:** Institute of Neuroscience/Newcastle University Institute for Ageing, Clinical Ageing Research Unit, Campus for Ageing and Vitality, Newcastle University, Newcastle upon Tyne, NE4 5PL UK

**Keywords:** Parkinson’s disease, Gait, Body worn monitor, Accelerometer, Free-living data, Ambulatory activity

## Abstract

**Background:**

Gait is emerging as a powerful diagnostic and prognostic tool, and as a surrogate marker of disease progression for Parkinson’s disease (PD). Accelerometer-based body worn monitors (BWMs) facilitate the measurement of gait in clinical environments. Moreover they have the potential to provide a more accurate reflection of gait in the home during habitual behaviours. Emerging research suggests that measurement of gait using BWMs is feasible but this has not been investigated in depth. The aims of this study were to explore (i) the impact of environment and (ii) ambulatory bout (AB) length on gait characteristics for discriminating between people with PD and age-matched controls.

**Methods:**

Fourteen clinically relevant gait characteristics organised in five domains (pace, variability, rhythm, asymmetry, postural control) were quantified using laboratory based and free-living data collected over 7 days using a BWM placed on the lower back in 47 PD participants and 50 controls.

**Results:**

Free-living data showed that both groups walked with decreased pace and increased variability, rhythm and asymmetry compared to walking in the laboratory setting. Four of the 14 gait characteristics measured in free-living conditions were significantly different between controls and people with PD compared to two measured in the laboratory. Between group differences depended on bout length and were more apparent during longer ABs. ABs ≤ 10s did not discriminate between groups. Medium to long ABs highlighted between-group significant differences for pace, rhythm and asymmetry. Longer ABs should therefore be taken into account when evaluating gait characteristics in free-living conditions.

**Conclusion:**

This study provides encouraging results to support the use of a single BWM for free-living gait evaluation in people with PD with potential for research and clinical application.

## Background

A more efficient healthcare system is required to cope with increased life expectancy and the growing world population where ageing related neurological diseases, gait disorders, and falls risk represent a major challenge and burden [1]. In this context quantitative measurement of gait has an important role to play to detect early disease and to inform disease mechanism and progression, and optimal management. Until recently, gait assessment has been limited to specialised laboratory facilities providing useful information about gait impairment in ageing and pathology such as Parkinson’s disease (PD) [2–5]. Body worn monitors (BWMs) [6] now provide a robust and inexpensive solution for continuous monitoring of ambulatory activity in both controlled [7–11] and free-living environments [3, 4, 12, 13].

In line with developments in gait measurement, frameworks to characterise gait are being developed which take a more comprehensive view by expanding the boundaries of measurement. Gait is conceptualised at micro level (i.e. spatiotemporal and gait dynamics), and macro level (i.e. volume, pattern and variability of ambulatory activity). Measuring gait in real life reflects habitual gait performance [14, 15], and is not confounded by heightened attention or altered by observer effect found during laboratory based assessment, even when naturalistic environments are simulated [16]. BWMs also allow movement to be captured continuously over longer periods of time which is not practical in a laboratory or clinical setting.

Even though the use of modern BWMs is increasing in both controlled and free-living environments, this is still a relatively new field of research where data extraction and analysis methods are constantly under development [17]. To date, important established gait characteristics such as stance time, swing time, and asymmetry gait characteristics have not yet been quantified in free-living contexts for people with PD or older adults, while several novel frequency based outcomes (although promising) currently lack a basis from which to interpret their clinical meaningfulness [2–5].

Quantifying gait characteristics in unsupervised environments presents considerable contextualising and methodological challenges. Despite these challenges, results from previous work [3–5, 18] have shown the potential benefits of data collected in unsupervised and uncontrolled conditions for detecting falls risk in people with PD and older adults. However, further research is required to test a more comprehensive range of gait characteristics than what has currently been achieved. Also, protocols for derivation of gait outcomes differ, making interpretation difficult [19]. For example, some studies evaluate gait characteristics by recording durations that range from three days to eight weeks [4, 20–23]. There is also methodological ambiguity surrounding the optimal bout length to use for extraction of gait characteristics. Ambulatory activity is made up of ambulatory bouts (ABs) of different lengths reflecting the context (home, community) and activity the individual is engaged in. It is likely that context and activity will impact on gait characteristics making bout length an important consideration. There is no clear definition of AB and arbitrary values are utilised across studies where criteria may vary based on number of steps or length of time [24]. For example, AB lengths ranging from three steps to longer than 60s have been used [2, 3, 12, 18, 23, 25, 26], even though it has been shown that adults tend to walk in short ABs (on average less than 30s with the highest percentage of ABs lasting 20s or less) [13, 18, 21]. Moreover some studies evaluating free-living gait characteristics recorded over three days in people with PD limited analysis to ABs longer than 60s only, although the authors did not address the reproducibility of laboratory versus free-living outcomes [3–5, 22].

The aims of this study were therefore to: (i) explore the impact of environment and pathology by analysing differences between people with PD and controls in the laboratory and free-living environments; (ii) investigate the impact of bout length on free-living gait characteristics for discriminating between groups. We carried out quantitative gait analysis in controlled and free-living environments using a theoretical model of gait to inform selection of gait characteristics and explored the influence of bout length on gait characteristics to discriminate between groups. We had two a-priori hypotheses:

i. between-group differences would be more apparent in free-living conditions than in the laboratory;
ii bout length would impact on free-living gait characteristics for both groups and influence between-group differences.

## Methods

### Participants

PD participants and controls were recruited from the Incidence of Cognitive Impairment in Cohorts with Longitudinal Evaluation—GAIT (ICICLE-GAIT) study. This is a collaborative study with ICICLE-PD, an incident cohort study (Incidence of Cognitive Impairment in Cohorts with Longitudinal Evaluation—Parkinson’s disease) conducted between June 2009 and December 2011 [27, 28].

Participants were excluded if they had a poor command of English and any neurological (other than PD), orthopaedic or cardiothoracic conditions that may have markedly affected their walking or safety during the testing sessions. In addition, PD participants had to be diagnosed with idiopathic PD according to the UK Parkinson’s Disease Brain Bank criteria and were excluded if they presented with significant memory impairment (Mini Mental State Exam (MMSE) < 24 [29]), dementia with Lewy bodies, drug induced parkinsonism, ‘vascular’ parkinsonism and atypical forms of parkinsonism such as progressive supranuclear palsy, multiple system atrophy, or corticobasal degeneration, according to accepted diagnostic criteria [30].

### Ethics, consent and permissions

Testing took place at the Clinical Ageing Research Unit, Newcastle University. This study was conducted according to the declaration of Helsinki and had ethical approval from the Newcastle and North Tyneside research ethics committee. All participants signed an informed consent form prior to testing.

### Demographic and clinical measures

Age and sex were recorded for each participant. The severity of PD motor symptoms was measured using the Hoehn and Yahr scale [31], which ranges from 0 (no symptoms) to 5 (wheelchair bound or bedridden if unaided) and section III of the modified Movement Disorder Society version of the Unified Parkinson’s Disease Rating Scale (MDS-UPDRS [32]), which ranges from 0 (no motor symptoms) to 132 (severe motor symptoms). Levodopa equivalent daily dose (LEDD) scores were calculated according to established methods [33].

### Laboratory data collection: equipment and gait protocol

**Fig. 1:**
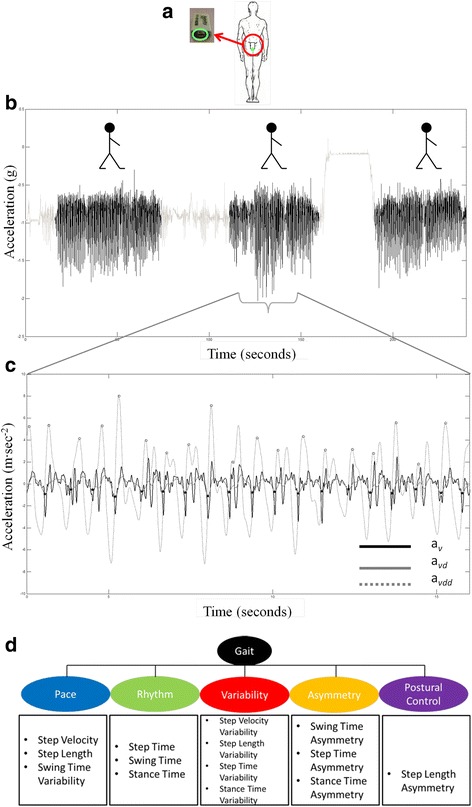
**a** Example of body worn monitor placement for both the laboratory based and free-living data collection. **b** *Vertical* acceleration and walking bout extraction (signal segments in *black*) from free-living data. **c** Example of gait characteristic extraction from walking bouts: detecting initial contacts (*black stars*) and final contacts (*white circles*). The *black solid line* represents vertical acceleration (a_v_), the *dashed line* represents the differentiated with Gaussian CWT of a_v_ (a_vd_), and the *dotted line* represents the differentiated with Gaussian CWT of a_vd_ (a_vdd_). **d** Conceptual model of gait representing domains and 14 gait characteristics

Each participant was asked to wear a single tri-axial accelerometer-based BWM (Axivity AX3, York, UK; dimensions: 23.0 × 32.5 × 7.6 mm; weight: 9 g; accuracy: 20 parts per million) which has been validated for its suitability in capturing high-resolution data akin to human movement [34]. The BWM was located on the fifth lumbar vertebra (L5, Fig. 1(a)), attached directly to the skin with double sided tape (Wig Tape, Natural Image, UK) and covered with Hypafix (BSN Medical Limited, Hull, UK). The device was programmed to capture data at 100 Hz (16-bit resolution) and at a range of ± 8 g [11].

Participants were asked to perform four intermittent straight line walking trials over a 10 m walkway at their preferred speed [14, 35, 36]. People with PD were tested approximately one hour after their medication intake.

### Free-living data collection: protocol

At the end of the laboratory testing session participants were asked to wear the BWM for one week [20]. The BWM was attached above L5 with a hydrogel adhesive (PALStickies, PAL Technologies, Glasgow, UK) and covered with the Hypafix bandage for extra support. The BWM was programmed to record continuously for 7 days. Participants were asked to continue their daily activities as usual and not to change their routine. Upon completion of recording, participants removed the device and posted it back to the researcher as detailed in previous work [13].

### Data processing and analysis

#### Data processing and variable extraction – laboratory

BWM data were downloaded to a computer, segmented into four different straight line passes using time stamps and analysed by a bespoke MATLAB® (R2012a) program. Accelerometer signals were transformed to a horizontal-vertical coordinate system [37], and filtered with a 4th order Butterworth filter at 20 Hz [7, 8, 38]. The calculation of the 14 gait characteristics representative of five domains (pace, variability, rhythm, asymmetry and postural control, Fig. 1(d)) is extensively described in [10, 11]; the same methodology was applied to both the groups. Briefly: the initial contact (IC, heel strike) and final contact (FC, toe-off) events within the gait cycle were identified from the Gaussian continuous wavelet transform of the vertical acceleration. ICs and FCs detection allowed the estimation of step, stance and swing time [11]. The IC events were also used to estimate step length using the inverted pendulum model [38]. To estimate a value for step velocity we utilised the simple ratio between step distance (length) and step time [11].

**Fig. 2:**
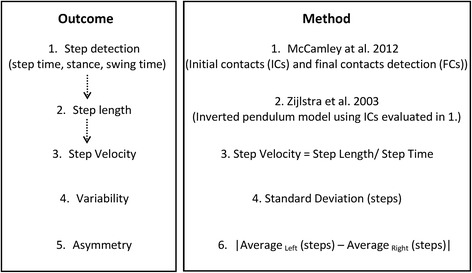
Summary flowchart of outcomes and methodology used for evaluation of the 14 gait characteristics of the gait model

From this it was possible to determine 14 gait characteristics of the theoretical model of gait which comprise 5 domains (pace, variability, rhythm, asymmetry and postural control) as detailed elsewhere [10, 11, 36]. To calculate step variability, the standard deviation (SD) from all steps (left and right combined) was calculated. Asymmetry was determined as the absolute difference between left and right steps (alternating) for each walking pass, averaged across all passes [11, 35, 39]. A summarising flowchart of this methodology is presented in Fig. 2.

#### Data processing and variable extraction – free-living data

Once the BWM was received, data were downloaded, segmented (per calendar day). For each day, individual ABs were extracted via MATLAB®, where a ‘bout’ was defined as the continuous length of time spent walking [13] (Fig. 1(b)). AB were detected applying selective thresholds on the standard deviation and the magnitude vector of the triaxial accelerations [40]. The 14 gait characteristics previously described [11] were evaluated from each of the detected AB (section 2.6.1, Fig. 1(c)).

### Data considerations

For the purpose of this study we decided to take a conservative approach and chose a threshold of three steps (minimum bout length) to define ABs [12, 18, 23, 26], with no threshold set for the maximum resting period between consecutive ABs [41]. Therefore each AB was considered individually in order to ensure robustness for the evaluation of the gait characteristics and to avoid sources of error in step detection, or for the calculation of variability and asymmetry characteristics (see section 2.6.1).

For consistency with the procedure used in the laboratory setting, the gait characteristics were evaluated for each single AB and then averaged over the 7 days.

### Statistical analysis

Statistical analysis was carried out using SPSS v19 (IBM). Normality of data was tested with a Shapiro-Wilk test. Descriptive statistics were reported as means and standard deviations (SD), or median and range depending on the normality of the distributions of gait characteristics. Clinical and demographic characteristics were described but not used in further analysis.

To test the impact of pathology and environment we examined between-group differences (controls vs. people with PD) using parametric t-tests or Mann–Whitney U tests (if not normally distributed) for each gait characteristic measured in the laboratory and in free-living conditions, and within-group difference (laboratory vs. free-living data) using Wilcoxon signed-rank tests. Spearman rank-order correlations and Mann–Whitney U tests were used to examine the agreement between laboratory based and free-living data.

Secondary analysis explored the impact of AB length on free-living gait characteristics with reference to pathology and environment. ABs were grouped depending on their length (ABs ≤ 10s, 10s < ABs ≤ 20s, 20s < ABs ≤ 30s, 30s < ABs ≤ 60s, 60s < ABs ≤ 120 s, ABs > 120 s) and comparisons of gait characteristics between PD and CL were performed for each length using independent parametric or Mann–Whitney U tests.

We used a threshold of *p* < 0.01 to guide statistical interpretation. Given the exploratory nature of this study, we did not correct for multiple comparisons [42, 43]. However, we provide the *p* value for each comparison so that the reader may assess the statistical strength of our findings.

## Results

Fifty controls and 47 people with PD were assessed. Compared to controls, people with PD were age matched but included proportionally less women (controls: 46 %, PD: 28 %), and presented with lower balance confidence; poorer cognition; and increased fatigue and depression (although the depression scores remained within the normal range). Participants with PD were in the early stages of the disease with mild motor symptoms. Participant demographic, clinical and cognitive descriptors are shown in Table 1.

**Table 1:** Demographic data.

Characteristic	CL (*n* = 50) Mean (SD)	PD (*n* = 47) Mean (SD)	*p*
Male/female (n)	m 27, f 23	m 34, f 13	0.062
Age (years)	69.8 (7.2)	69.1 (8.3)	0.694
MMSE (0 - 30)	28.4 (1.7)	28.3 (2.0)	0.827
MoCA (0 - 30)^+^	27.6 (2.4)	26.0 (3.8)	**0.016**
GDS (0 - 15)	1.0 (1.5)	2.7 (2.7)	**<0.001**
MFI Total fatigue (20 - 100)	34.7 (13.2)	49.9 (18.6)	**<0.001**
ABCs (0 - 100 %)	91.7 (11.0)	80.4 (18.4)	**0.001**
Hoehn & Yahr stage (n)	-	HY I 5	-
		HY II 39	
		HY III 3	
Levodopa Equivalent Daily Dose	-	419.6 (214.0)	-
MDS-UPDRS III	-	32.0 (10.1)	-
Freezing of gait (n, %)	-	7 (14.9 %)	-
Motor Phenotype (n)	-	PIGD 18	-
		ID 6	
		TD 23	

*MMSE* mini mental state exam, *MoCA* montreal cognitive assessment, *GDS* geriatric depression scale, *MFI* multidimensional fatigue inventory, *ABCs* activities specific balance confidence scale, *UPDRS* Unified Parkinson’s disease rating scale, *PIGD* postural instability and gait disorder phenotype, *ID* indeterminate phenotype, *TD* tremor dominant phenotype. *p* difference between CL and people with PD. In bold significant *p* values (*p* < 0.05)

Clinical and demographic characteristics for control participants (CL), and people with Parkinson’s disease (PD). In bold are shown significant *p* values < 0.05

### Impact of environment and pathology

**Fig. 3:**
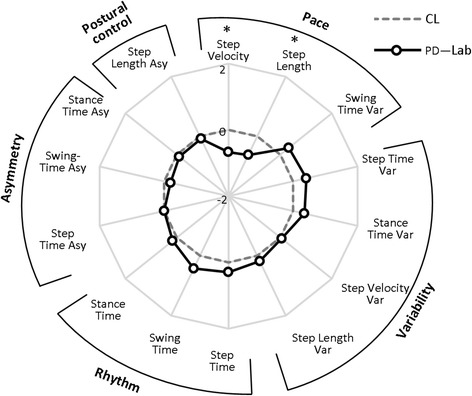
Radar plot illustrating the 14 gait characteristics organised by domain for people with Parkinson’s disease (PD) and controls (CL) evaluated in the laboratory (Lab). The central dotted line represents CL data, deviation from zero along the axis radiating from the centre of the plot represents how many standard deviations (range: ±2 SD, *z* score based on control means and standard deviations) the PD differ from CL. Asterisks represent significant differences between PD and CL (*p* values < 0.01)

**Fig. 4:**
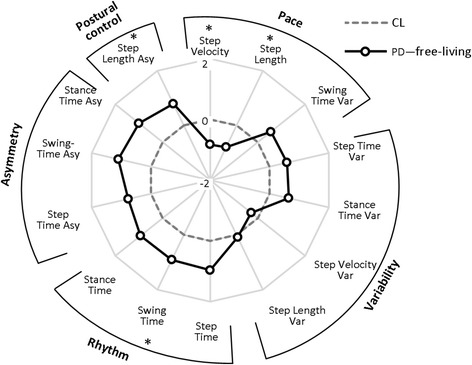
Radar plot illustrating the 14 gait characteristics organised by domain for people with Parkinson’s disease (PD) and controls (CL) evaluated in free-living conditions. The central *dotted line* represents CL data, deviation from zero along the axis radiating from the centre of the plot represents how many standard deviations (range: ± 2 SD, *z* score based on control means and standard deviations) the PD differ from CL. *Asterisks* represent significant differences between PD and CL (*p* values < 0.01)

Fourteen gait characteristics were replicated in both laboratory and free-living conditions (Table 2). Not surprisingly the impact of environment was significant for all gait characteristics (*p* < 0.001). Both groups walked with decreased pace, increased rhythm, higher variability and asymmetry in free-living compared to the laboratory environment (Table 2). Free-living data showed low to moderate correlations (*r* ≤ 0.453) with laboratory results for both groups. In both environments people with PD walked at a slower pace (with slower and shorter steps), with increased rhythm and a more variable and asymmetric walking pattern with respect to controls (Table 2, Figs. 3 and 4). Between-group differences in gait characteristics were exaggerated in free-living conditions (Fig. 4). Laboratory based results showed significant between-group differences for two/14 gait characteristics (step velocity and step length) representing pace (Table 2, Fig. 3). This increased to four/14 gait characteristics comprising pace, rhythm and variability in free-living conditions (Table 2, Fig. 4).

**Table 2:** Laboratory based and free-living gait characteristics.

Domain/gait characteristic	BWM Lab			BWM free-living		
	CL (*n* = 50)	PD (*n* = 47)	*p*	CL (*n* = 50)	PD (*n* = 47)	*p*
Pace						
Step Velocity (m/s)	1.393 ± 0.207	1.254 ± 0.211	**0.002**	1.097 (0.48)	1.017 (0.426)	**<0.001**
Step Length (m)	0.726 ± 0.095	0.667 ± 0.073	**0.001**	0.601 (0.183)	0.578 (0.243)	**<0.001**
Swing Time Var (s)	0.018 (0.113)	0.025 (0.103)	0.051	0.147 (0.125)	0.151 (0.134)	0.014
Variability (SD)						
Step Velocity Var (m/s)	0.073 (0.301)	0.081 (0.223)	0.253	0.383 (0.494)	0.362 (0.221)	0.070
Step Length Var (m)	0.033 (0.096)	0.039 (0.094)	0.050	0.151 (0.079)	0.152 (0.091)	0.660
Step Time Var (s)	0.019 (0.109)	0.028 (0.085)	0.037	0.175 (0.156)	0.181 (0.179)	0.037
Stance Time Var (s)	0.022 (0.109)	0.029 (0.092)	0.088	0.188 (0.161)	0.196 (0.249)	0.034
Rhythm						
Step Time (s)	0.525 ± 0.047	0.539 ± 0.058	0.206	0.593 (0.144)	0.605 (0.318)	0.017
Swing Time (s)	0.371 ± 0.040	0.388 ± 0.055	0.092	0.449 (0.113)	0.458 (0.252)	**0.008**
Stance Time (s)	0.679 ± 0.061	0.689 ± 0.069	0.450	0.741 (0.166)	0.756 (0.434)	0.035
Asymmetry						
Step Time Asy (s)	0.007 (0.140)	0.009 (0.057)	0.268	0.093 (0.086)	0.098 (0.142)	0.116
Swing Time Asy (s)	0.010 (0.126)	0.007 (0.055)	0.473	0.084 (0.064)	0.091 (0.133)	0.013
Stance Time Asy (s)	0.007 (0.140)	0.006 (0.035)	0.665	0.094 (0.086)	0.100 (0.131)	0.097
Postural Control						
Step Length Asy (m)	0.007 (0.060)	0.009 (0.086)	0.845	0.081 (0.043)	0.088 (0.070)	**0.004**

*Var* Variability, *Asy* Asymmetry

Values of gait characteristics for controls (CL) and people with Parkinson’s disease (PD) for laboratory based data (BWM Lab) and averaged free-living data (BWM free-living), values of normal gait characteristics are presented as mean ± standard deviation (SD), non-normal as median (range). Results of the *t*-test or the Mann–Whitney *U* test (for non-normal gait characteristics) analysis between people with PD and CL are reported, in bold are shown *p* values < 0.01

### Impact of bout length and pathology

**Fig. 5:**
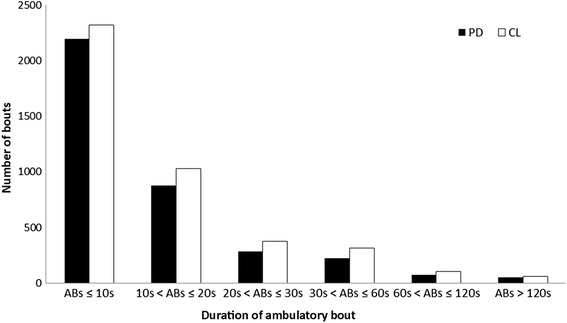
Mean number of walking bouts over seven days of recording for different ambulatory bout (AB) lengths (ABs ≤ 10s, 10s < ABs ≤ 20s, 20s < ABs ≤ 30s, 30s < ABs ≤ 60s, 60s < ABs ≤ 120 s, ABs > 120 s) for both people with Parkinson’s disease (PD, *black*) and controls (CL, *white*)

**Fig. 6:**
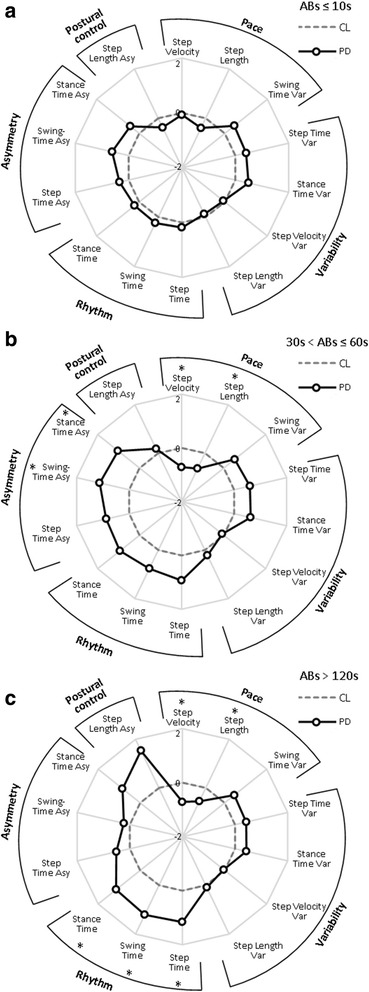
Radar plot illustrating the 14 gait characteristics organised by domain for people with Parkinson’s disease (PD) and controls (CL) evaluated in free-living conditions for ambulatory bouts (ABs) ≤ 10s (panel (**a**)), 30s < ABs ≤ 60s (panel (**b**)), and ABs > 120 s (panel (**c**)). The central dotted line represents CL data, deviation from zero along the axis radiating from the centre of the plot represent how many standard deviations (range: ± 2 SD, *z* score based for each bout length on control means and standard deviations) the PD differ from CL. Asterisks represent significant differences between PD and CL (*p* values < 0.01)

The majority of ABs were shorter than 10s for both groups (control: 55 %, PD: 59 %), with only 3 % lasting longer than 60s for both the groups. All participants performed at least two ABs over 120 s (a mean of 52 for people with PD and 62 for CL), however these longer bouts represented only 1 % of the total number of ABs for both groups (Fig. 5). For both groups pace increased with longer bouts, rhythm tended to increase for bouts lasting 30s and then decreased for longer bouts, variability and asymmetry decreased with the increase of AB duration, and asymmetry approached similar values observed in the laboratory for bouts longer than 120 s (Table 3). Between-group differences were influenced by bout length (Table 3, Fig. 6). For the shortest ABs (≤10s) there were no differences (Fig. 5(a)), for ABs between 10s - 20s (step length) and 20s - 30s (step length and step velocity) only pace differed between groups. ABs between 30s - 60s showed that people with PD walked with significantly slower pace and increased asymmetry compared to controls (Fig. 6(b)). For ABs between 60s - 120 s, PD participants demonstrated slower pace than controls. ABs longer than 120 s showed that five/14 gait characteristics comprising pace and rhythm differed between groups (Fig. 6(c)).

**Table 3:** Impact of bout length on free-living gait characteristics

Domain/gait characteristic	ABs ≤ 10s			10s < ABs ≤ 20s			20s < ABs ≤ 30s		
	CL (*n* = 50)	PD (*n* = 47)	*p*	CL (*n* = 50)	PD (*n* = 47)	*p*	CL (*n* = 50)	PD (*n* = 47)	*p*
Pace	1663 steps	1603 steps		2196 steps	1926 steps		1517 steps	1181 steps	
Step Velocity (m/s)	0.934 (0.374)	0.910 (0.640)	0.145	1.046 ± 0.067	1.010 ± 0.111	0.059	1.082 (0.385)	1.009 (0.569)	**0.003**
Step Length (m)	0.537 (0.143)	0.528 (0.208)	0.066	0.588 (0.149)	0.570 (0.198)	**0.002**	0.600 (0.160)	0.587 (0.164)	**0.001**
Swing Time Var (s)	0.166 ± 0.013	0.172 ± 0.021	0.097	0.156 (0.093)	0.162 (0.097)	0.056	0.152 (0.133)	0.158 (0.097)	0.056
Variability (SD)									
Step Velocity Var (m/s)	0.390 (0.172)	0.374 (0.232)	0.196	0.383 (0.212)	0.376 (0.221)	0.479	0.388 (0.347)	0.375 (0.199)	0.511
Step Length Var (m)	0.163 (0.048)	0.160 (0.072)	0.231	0.153 (0.057)	0.154 (0.065)	0.431	0.150 (0.086)	0.154 (0.052)	0.048
Step Time Var (s)	0.204 (0.095)	0.205 (0.138)	0.457	0.186 (0.116)	0.187 (0.155)	0.289	0.181 (0.172)	0.183 (0.155)	0.201
Stance Time Var (s)	0.217 (0.114)	0.219 (0.193)	0.431	0.198 (0.144)	0.201 (0.191)	0.191	0.192 (0.194)	0.195 (0.194)	0.164
Rhythm									
Step Time (s)	0.611 (0.106)	0.61 (0.306)	0.639	0.609 (0.121)	0.618 (0.384)	0.220	0.607 (0.140)	0.619 (0.407)	0.054
Swing Time (s)	0.468 (0.090)	0.471 (0.230)	0.436	0.467 (0.103)	0.474 (0.298)	0.201	0.463 (0.105)	0.477 (0.305)	0.033
Stance Time (s)	0.760 (0.159)	0.755 (0.403)	0.891	0.761 (0.186)	0.760 (0.512)	0.488	0.753 (0.193)	0.758 (0.553)	0.130
Asymmetry									
Step Time Asy (s)	0.164 (0.123)	0.164 (0.171)	0.639	0.083 (0.135)	0.084 (0.156)	0.751	0.056 (0.103)	0.058 (0.112)	0.402
Swing Time Asy (s)	0.123 (0.080)	0.125 (0.133)	0.316	0.075 (0.116)	0.077 (0.144)	0.164	0.049 (0.082)	0.053 (0.147)	0.151
Stance Time Asy (s)	0.165 (0.116)	0.164 (0.149)	0.702	0.084 (0.131)	0.084 (0.149)	0.398	0.055 (0.108)	0.059 (0.132)	0.217
Postural Control									
Step Length Asy (m)	0.125 ± 0.012	0.121 ± 0.018	0.173	0.090 (0.117)	0.088 (0.102)	0.359	0.073 (0.068)	0.069 (0.074)	0.071
	30s < ABs ≤ 60s			60s < ABs ≤ 120 s			ABs > 120 s		
	CL (*n* = 50)	PD (*n* = 47)	*p*	CL (*n* = 50)	PD (*n* = 47)	*p*	CL (*n* = 50)	PD (*n* = 47)	*p*
Pace	2226 steps	1632 steps		1596 steps	1205 steps		3797 steps	2975 steps	
Step Velocity (m/s)	1.103 (0.411)	1.038 (0.422)	**<0.001**	1.110 (0.419)	1.032 (0.472)	**0.003**	1.137 (1.035)	1.029 (0.686)	**<0.001**
Step Length (m)	0.609 (0.173)	0.593 (0.185)	**0.003**	0.608 (0.194)	0.590 (0.236)	0.034	0.632 (0.269)	0.581 (0.206)	**0.005**
Swing Time Var (s)	0.147 (0.138)	0.153 (0.134)	0.029	0.142 (0.127)	0.144 (0.156)	0.279	0.125 (0.174)	0.146 (0.181)	0.014
Variability (SD)									
Step Velocity Var (m/s)	0.376 (0.326)	0.369 (0.265)	0.411	0.370 ± 0.051	0.353 ± 0.062	0.153	0.354 (0.559)	0.351 (0.803)	0.573
Step Length Var (m)	0.148 (0.106)	0.150 (0.093)	0.141	0.144 (0.073)	0.146 (0.115)	0.806	0.145 (0.153)	0.150 (0.126)	0.511
Step Time Var (s)	0.174 (0.173)	0.178 (0.178)	0.033	0.170 (0.168)	0.172 (0.219)	0.214	0.151 (0.224)	0.180 (0.233)	0.042
Stance Time Var (s)	0.187 (0.199)	0.190 (0.249)	0.054	0.182 (0.185)	0.187 (0.297)	0.179	0.160 ± 0.047	0.181 ± 0.051	0.042
Rhythm									
Step Time (s)	0.600 (0.092)	0.610 (0.333)	0.057	0.591 (0.118)	0.604 (0.292)	0.033	0.576 (0.253)	0.599 (0.570)	**0.001**
Swing Time (s)	0.457 (0.100)	0.469 (0.259)	0.033	0.443 (0.110)	0.453 (0.191)	0.069	0.425 (0.132)	0.443 (0.403)	**<0.001**
Stance Time (s)	0.750 (0.128)	0.753 (0.448)	0.145	0.742 (0.139)	0.755 (0.341)	0.041	0.723 (0.310)	0.754 (0.666)	**0.001**
Asymmetry									
Step Time Asy (s)	0.041 (0.067)	0.045 (0.068)	0.012	0.029 (0.034)	0.031 (0.078)	0.082	0.019 (0.037)	0.022 (0.17)	0.419
Swing Time Asy (s)	0.036 (0.055)	0.041 (0.072)	**0.001**	0.026 (0.033)	0.029 (0.059)	0.166	0.018 (0.050)	0.019 (0.127)	0.525
Stance Time Asy (s)	0.041 (0.067)	0.045 (0.071)	**0.009**	0.028 (0.041)	0.029 (0.043)	0.065	0.019 (0.040)	0.020 (0.211)	0.453
Postural Control									
Step Length Asy (m)	0.055 (0.064)	0.055 (0.073)	0.554	0.036 (0.047)	0.036 (0.058)	0.868	0.020 (0.069)	0.024 (0.766)	0.073

*Var* variability, *Asy* asymmetry, *steps* steps per day

Values of gait characteristics for controls (CL) and people with Parkinson’s disease (PD) derived from free-living data grouped by ambulatory bout (AB) lengths (ABs ≤ 10s, 10s < ABs ≤ 20s, 20s < ABs ≤ 30s, 30s < ABs ≤ 60s, 60s < ABs ≤ 120 s, ABs > 120 s), values of normal gait characteristics are presented as mean ± standard deviation (SD), non-normal as median (range). Average number of steps per day (steps) taken into account for each AB length are presented for both the groups. Results of the t-test or the Mann–Whitney *U* test (for non-normal gait characteristics) analysis between people with PD and CL are reported, in bold are shown *p* values < 0.01

## Discussion

To our knowledge this is the first study to quantify a comprehensive range of clinically relevant gait characteristics in a large cohort of controls and people with PD (total *n* = 97) in laboratory and free-living conditions. We found that free-living conditions heightened between-group differences. Secondary findings were that bout length had an impact on gait characteristics and long ABs were more discriminative of PD-specific gait impairment than short ABs.

### Impact of environment and pathology

Regardless of pathology, compared to laboratory based data, free-living conditions attenuated gait performance. Although direct comparison between laboratory based and totally unsupervised free-living gait characteristics in people with PD has not been previously reported, these results support early work by Weiss et al. [3, 22] and Brodie et al. [23] who found a trend towards higher variability (frequency [22] or step time [23] measures) and lower cadence [23] for older adults in free-living condition compared to laboratory settings. This could be due to the fact that (a) participants may alter their gait by increasing their pace and decreasing their rhythm while under observation in controlled environments [16, 23], and (b) the BWM seems to be a more sensitive tool (i.e. higher values compared to laboratory reference results (e.g. instrumented walkway)) in evaluating asymmetry and variability gait characteristics not only in the laboratory setting but also in real-life conditions [11].

As expected our findings suggest between-group differences in gait characteristics were exaggerated for gait measured in free-living contexts. Although still unclear, sensitivity of free-living data to pathology may be explained partly by the reduction of cognitive (attentional) input which is required for optimal gait in people with PD [44, 45]*,* and impaired under dual task conditions [46]. Free-living gait is naturalistically dual task because of the distractions, environmental obstacles, and task complexities that limit attentional compensation; while conversely attentional control is optimised during scripted gait tests in the laboratory [16, 47]. Gait measured in free-living contexts may therefore be a more sensitive surrogate marker of PD pathology compared with laboratory based measurements and be superior in assessing features of the disease such as heightened falls risk and freezing of gait [3–5].

### Impact of bout length and pathology

In free-living conditions both groups performed a large number of very short ABs (ABs ≤ 10s) rather than prolonged ABs [18, 21, 48] most likely reflecting habitual behaviours and moving in a constrained environment such as a house. Consistent with our hypothesis, gait characteristics of people with PD and controls changed with respect to bout length and approximated laboratory values for prolonged ABs (> 120 s) where the time was closer to laboratory testing protocol. This suggests that gait performance depends on AB length, moreover gait characteristics and the impact of pathology vary as a function of AB length. Indeed specifically gait impairment in people with PD was only evident when looking at longer ABs, with no group differences observed during very short AB (≤ 10s). Between-group differences in asymmetry were found for medium length ABs but not for prolonged ABs, while variability was more evident for longer ABs. Being able to detect these changes is important because asymmetry represents a primary feature of a number of neurological disorders such as PD [36, 49]. We speculate ABs between 30s and 60s could represent walking indoors (e.g. home, shopping centre, etc.) where increased change of directions, turning, dual tasking, and the environment itself could affect the asymmetry of walking, while prolonged ABs (> 120 s) could correspond to walking outdoors (e.g. park) so that a regular steady state is more likely to be achieved. These results suggest that gait measured in the free-living context sensitises measurement of pathology reflecting the heightened control challenges and limited compensatory adaptability.

### Implications for free-living data analysis

Results from this study have important implications for analysis of gait in free-living data. Very short ABs (ABs ≤ 10s) did not discriminate for pathology in this instance, suggesting that a minimum of 10s is required to detect changes in mean and asymmetry gait characteristics in people with PD. Only bouts of medium length (30s < ABs ≤ 60s) were able to detect between-group differences for asymmetry. Moreover only 3 % of the walking bouts were greater than 60s. Therefore when considering free-living data it is essential to take into account ABs longer than 10s because ABs longer than 60s represent only a partial picture of gait performance.

### Limitations

This study informs understanding of the effect of bout length on outcomes, however further work is required to identify the merits in merging short ABs to provide more meaningful data [41].

We used pooled intermittent walks collected in the lab which can be comparable in duration to short-medium ABs (10s - 30s) collected in free-living environments, in the future longer walks (e.g. two minute walks) collected in the laboratory may be useful for comparing longer ABs (60-120 s).

Discriminating purposeful from non-purposeful walking bouts in constrained environments such as a home is challenging, and requires greater consideration.

This study did not set out to provide an interpretation of the data by revealing the context in which gait was performed. However, moving forward this will be important. Use of simultaneous video recording may be a solution although privacy issues then become evident. Moreover the effect of medication intake on fluctuations of gait in people with PD needs to be investigated and on/off periods will likely have an impact on gait characteristics. Lastly, despite choosing a stringent *p* value of 0.01, we acknowledge this does not completely mitigate the inflation of type I error introduced by multiple comparisons. We feel this approach is justified given the exploratory nature of the work as we did not want to unduly increase the risk of type II statistical error [42, 43]. Although the resulting findings will be important for future hypothesis generation, as noted they may vary with respect to the choice of correcting for multiple comparisons. Therefore, we recommend caution when applying our findings until they are replicated. To help the reader interpret the strength of the findings, we have included the full *p* values in Tables 2 and 3.

## Conclusions

In conclusion this study supports the use of a single BWM to quantify clinically relevant, pathology-sensitive gait characteristics in free-living environments. Results from this study provide a platform for future research to adopt a broader application of accelerometry data that will inform our understanding of gait in naturalistic environments, and the features associated with that performance.

## Abbreviations

AB: ambulatory bout
BWM: body worn monitor
CL: controls
FC: final contact
H&Y: Hoehn & Yahr Stage
Hz: Hertz
IC: initial contact
ICICLE-GAIT: incidence of cognitive impairment in cohorts with longitudinal evaluation-GAIT
L5: fifth lumbar vertebra
LEDD: levodopa equivalent daily dose
MDS-UPDRS: movement disorder society version of the unified parkinson’s disease rating scale
MMSE: mini mental state exam
PD: Parkinson’s disease
s: seconds
SD: standard deviation
